# Immune related biomarkers for cancer metastasis to the brain

**DOI:** 10.1186/s40164-022-00349-z

**Published:** 2022-12-16

**Authors:** Wei-Wei Chen, Timothy Shun Man Chu, LiangLiang Xu, Cai-Ning Zhao, Wai-Sang Poon, Gilberto Ka-Kit Leung, Feng-Ming (Spring) Kong

**Affiliations:** 1 Department of Clinical Oncology, Li Ka Shing Faculty of Medicine, University of Hong Kong, Pokfulam, Hong Kong, SAR China; 2 Royal Victoria Infirmary, Newcastle Upon Tyne Hospitals NHS Foundation Trust, Queen Victoria Road, Newcastle Upon Tyne, NE1 4LP UK; 3 Faculty of Medical Sciences, Newcastle University, Newcastle Upon Tyne, NE1 7RU UK; 4 Department of Clinical Oncology, The University of Hong Kong-Shenzhen Hospital, Shenzhen, China; 5 Neuro-Medical Center, The University of Hong Kong-Shenzhen Hospital, Shenzhen, China; 6 Department of Surgery, School of Clinical Medicine,LKS Faculty of Medicine, University of Hong Kong, Pokfulam, Hong Kong, SAR China

**Keywords:** Brain metastasis, Immune biomarkers, Tumor immune microenvironment, Systemic tumor immune environment

## Abstract

Brain metastasis accounts for a large number of cancer-related deaths. The host immune system, involved at each step of the metastatic cascade, plays an important role in both the initiation of the brain metastasis and their treatment responses to various modalities, through either local and or systemic effect. However, few reliable immune biomarkers have been identified in predicting the development and the treatment outcome in patients with cancer brain metastasis. Here, we provide a focused perspective of immune related biomarkers for cancer metastasis to the brain and a thorough discussion of the potential utilization of specific biomarkers such as tumor mutation burden (TMB), genetic markers, circulating and tumor-infiltrating immune cells, cytokines, in predicting the brain disease progression and regression after therapeutic intervention. We hope to inspire the field to extend the research and establish practical guidelines for developing and validating immune related biomarkers to provide personalized treatment and improve treatment outcomes in patients with metastatic brain cancers.

**Supplementary Information:**

The online version contains supplementary material available at 10.1186/s40164-022-00349-z.

## Introduction

Brain metastasis is a common cause of morbidity and mortality in cancer patients [1, 2]. The overall annual incidence amongst cancer brain metastasis patients ranges from 9–50% and varies greatly by primary cancer origin [3]. The estimated cases of brain metastasis were 150,000 to 200,000 all around the world, 98,000 to 170,000 in United States each year [4, 5]. The overall 2-year and 5-year survival rates of brain metastasis patients were 8.1% and 2.4% across all primary tumors [6]. The most common primary sites for brain metastasis are lung (39–56%), breast (13–30%), and melanoma (8–11%) [7], with overall median survival ranging from 3–14 [8], 5–34 to 3–17 months [9], respectively. Although significant progress has been made on the field, biomarker-guided precision treatment either for patient selection or enrichment is largely limited in brain metastasis.

The biological process of cancer brain metastasis is complicated. The crosstalk between cancer and host immune cells in the local tumor immune microenvironment (TIME) [10, 11] is a critical part of this complexity during brain metastasis formation. The tumor cells are also influenced by the systemic immunity of the host [12], recently named as Systemic Immune Environment (STIE) [13], which is in constant interaction with the TIME. The lymphatic and blood circulation system serves as conduit between TIME and STIE, which carries immune cells and immune modulating factors [14]. The functional immune units including the regulator and effector immune cells, the various immune modulating molecules such as chemokines/cytokines, and the expression profiles of immune checkpoints of other cells and the heterogenous tumor mutation burdens, represent the current key research areas with exciting developments [15, 16]. Thus, the level of these factors reflects the host immune status in patients with brain metastasis at baseline or follow-up and acts as indicators of the pathogenic processes [17] or responses to an intervention [18], can be used as biomarkers.

Here we will review the biological functions and clinical significance of cell and molecular biomarkers of both TIME including the primary tumor and the brain metastatic tumors, and STIE including the cerebrospinal fluid (CSF) and blood during brain metastasis. Of the cell biomarkers, we will focus our review on the immune cells subtypes such as T lymphocytes, B lymphocytes, myeloid-derived suppressive cells (MDSCs), macrophages and microglia [16, 19]. For molecular modulating factors, this review covers chemokines, cytokine, immune checkpoints [20] and the tumor mutational landscapes [21]. Furthermore, we will summarize the challenges and limitations of these biomarkers aiming to develop research strategies to advance.

## Overall role of the immune system in brain metastasis

Brain metastasis is a multi-step bi-directional process, where cancer cells interact with the immune system and influence each other, both locally and systemically [22]. The important first step of metastasis is when tumor cells enter and survive in the circulating system (Additional file 1). This is then followed by extravasation and infiltration of tumor cells into the brain by passing through the blood brain barrier (BBB) and proliferate in the brain microenvironment that is “protected” by BBB. During this process, these tumor cells have to evade the immune surveillance, typically through disturbing antigen recognition processes by genetic alterations, suppressing cytokine production, inducing the apoptosis of immune cells, stimulating the generation of regulatory T cells (Tregs), and expanding the MDSCs [23]. Meanwhile, the immune cells in peripheral system, lymphoid tissues and brain metastasis regions respond to these signals released by the metastasis tumor cells [24, 25]. These tumors associated immune cells are considered as a selected population of immune cells with specific immunological reactivity against brain metastatic tumor cells [26]. Thus, the immunological activity of these tumor-associated immune cells can reflect the local or systemic immune responses to the metastatic tumor cells, providing potential predictive and prognostic values. Furthermore, the immune responses to cancer brain metastasis are mediated not only by cell–cell interactions, but also by the coordinated actions of a diverse set of immune modulators including checkpoints, cytokines and metabolites from the TIME and STIE [27] (Additional file 1).

### Biomarkers associated immune perturbations in the TIME

#### Biomarkers associated with TIME features of the primary tumor

Before the initiation of brain metastasis, the malignant cells must be aggressive enough to escape the local TIME system at the primary system, which is normally associated with a series of mutations process to allow themselves survive under the immune election process and determine their fates which organs to metastasize [28]. These genetic mutations shield the tumor cells from the attacks by the cytotoxic immune cells by inducing the expression of co-immune inhibitory receptors and neoantigens [29, 30], and can be measured by tumor mutation burden (TMB) from the primary tumor or circulating tumor DNA, serving as biomarkers of immune relevance. Indeed, most patients with brain metastasis harbor higher TMBs, compared with the patients without brain metastasis reported in a clinical evidence analysis [31]. Mutations of the immune related genes are also seen. For example, higher amplification frequencies of MYC, YAP1, MMP13 and more frequent deletions in CDKN2A/B increased the incidence of brain metastasis in lung adenocarcinoma brain metastasis patient-derived xenograft mouse models [32].

Molecular immune modulators like PD-L1 expression in the primary tumor cells of melanoma was associated with a shorter overall survival in patients with brain metastasis, according to a retrospective study of 233 patients with brain metastasis and 111 paired primaries [33]. Moreover, patients with mutated genes in epidermal growth factor receptor (EGFR), cytotoxic T-lymphocyte associated protein 4 (CTLA4), PDCD1LG2, or ZEB1 genes complemented with lower PD-L1 protein expression were reported to have worse prognostic outcomes in patients with after surgical treatment for brain metastases [34]. Patients with high CXCL12 (C-X3-C Motif Chemokine Ligand 12) expression in their primary non-small cell lung cancer (NSCLC) also have a higher risk of developing brain metastases [35].

#### Biomarkers associated with TIME features of the metastatic brain lesions

The genetic features of the metastatic tumors, such as transcriptome expressions in the primary tumors can identify patients who are more susceptible to the progression of brain metastasis [29, 36]. The expression of phosphorylated signal transducer and activator of transcription 3 (Stat3), an important immune regulator gene, has been shown to be higher in melanoma brain-metastases relative to distant metastases to the rest of the body [37]. The activation of Stat3 is upregulated in human brain metastatic cells and contributes to brain metastasis of melanoma [32, 38].

Beyond the predictive roles of mutations in brain metastasis, these data also suggest a possible therapeutic intervention tailored to the genomics related treatments for patients with high risk for brain metastasis by profiling the genome change of these patients. However, the genomic variations that underlie the brain metastasis are generally complex, and often from studies with smaller sample sizes and hence smaller predictive power [39]. Therefore, it is necessary for us to balance our ability to measure multiple parameters of the immune response with the recognition of multiple interactions between cell types, cytokines and molecular networks to determine clinical outcomes and therapeutic responses. A recent study from brain metastatic 231-BR cells identified eight molecules that had prognostic values in patients with breast cancer with brain metastasis. Four of them (KRT19, FKBP10, GSK3B and SPANXB1) had a correlation with the infiltration of major immune cells in the brain TIME [40]. Nevertheless, more preclinical studies and patient trials are required to understand the features of primary tumors with brain metastasis and various brain metastatic lesions from similar primary tumors, which can help understanding the true nature of tumor-immune interaction in both primary and brain metastatic sites.

## STIE peripheral immune biomarkers in the circulation

**Fig. 1:**
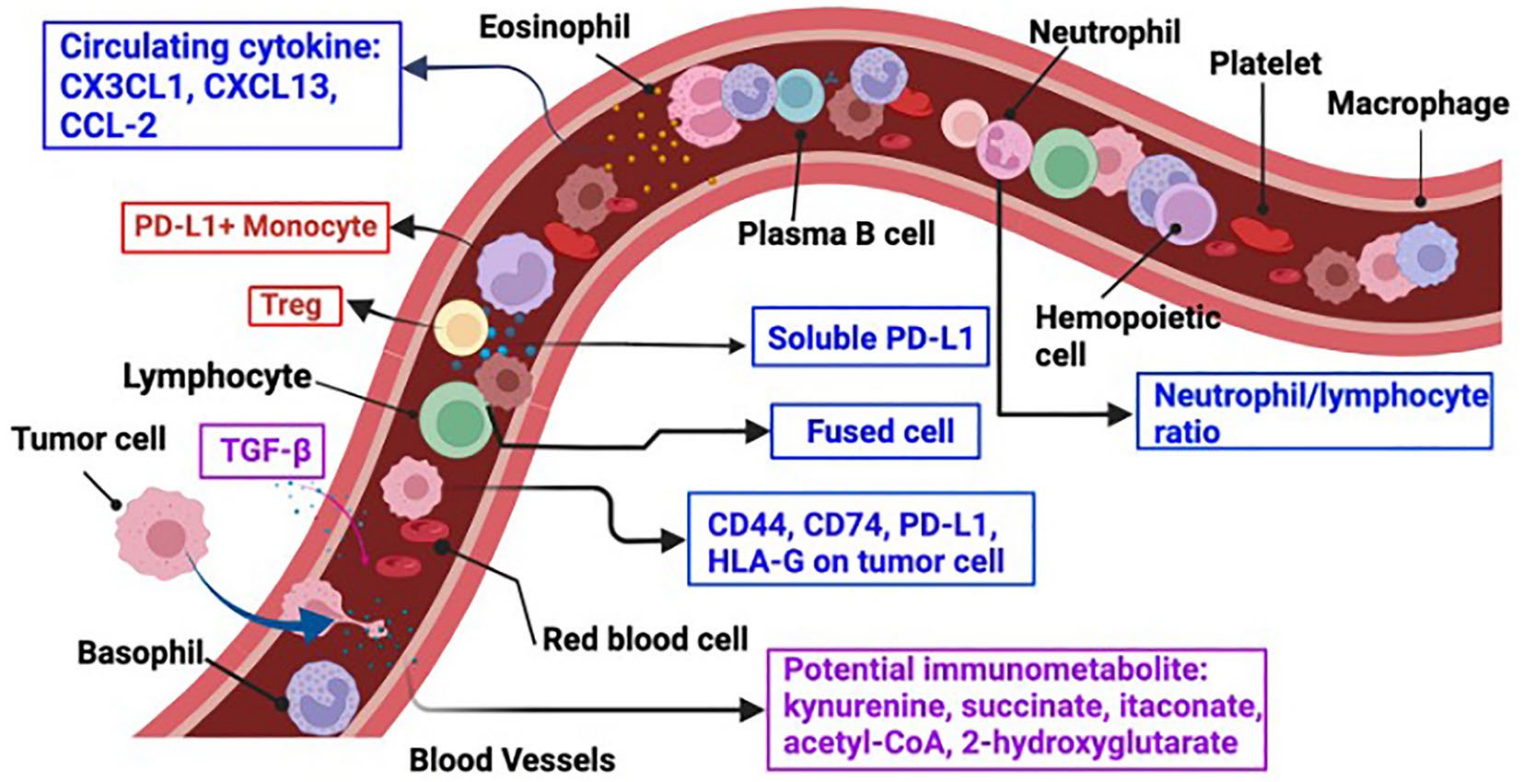
Illustration of STIE Potential Immune Biomarkers. Published biomarkers are shown. Red colored are the ones associated with poor survival. Blue colored are the risk factors of brain metastasis. Purple colored are the potential biomarkers for brain metastasis. *STIE* Systemic Tumor Immune Environment, *HLA-G* human leukocyte antigen G, *TGF-β* transforming growth factor beta, *Fused cells* immune cells fused with tumor cells, *Treg* Regulatory T cells

After metastasizing from the primary tumor sites, the tumor cells need to survive in the circulatory system (mainly in the blood), where these tumor cells will be spatiotemporally interacting with the immune components [41]. These interactions and immune perturbations caused by the tumor cells may also act as promising biomarkers for brain metastasis in the STIE [31] (Fig. 1).

### Immune checkpoints and ligands in the circulation STIE

Once metastatic tumor cells break free from the primary tumor and enter circulation, they interacts with the immune components in the blood. Circulating tumor cells (CTCs) will recruit macrophages to protect CTCs from being eradicated by the cytotoxic killer cells by eliciting secretion of various cytokines [42]. Notably, chitinase-3-like-1 (CHI3L1), the cytokine/growth factor released by the CTCs, was found to be correlated to metastasis and a dismal prognosis [43, 44]. In a breast cancer brain metastasis cohort of 40 patients, the expression of CD44 (3/40) and CD74 (39/40) found on CTCs were likely associated with the brain metastasis [45]. Programmed death ligand 1(PD-L1)^+^ CTCs were detected in 68.8% (11/16) patients with HR^+^HER-2 breast cancer [46]. Similar to PD-L1, the expression of the human leukocyte antigen-G (HLA-G) on tumor cells has been observed in various malignant tumors and also promote the progression of brain metastasis [47]. A study of 43 patients with brain metastasis revealed soluble PD-L1 in the plasma of some patients with melanoma (2/4), breast (3/29), lung cancer (3/29) and renal cell carcinoma brain metastasis patients (0/6) [48]. Another study of 44 brain metastasis patients found that the soluble PD-L1 was detected in 7/44 (15.9%) of patients [49]. A study of 90 patients with small cell lung cancer reported increased PD-L1 level in patients with brain metastasis [50]. Studies with larger sample sizes and clinical outcome correlates are needed to further explore the possibilities of using blood biomarkers for checkpoint inhibitors.

### Immunomodulating factors such as cytokines in the circulation STIE

Serum from patients with brain metastases showed that CX3CL1 (C-X3-C Motif Chemokine Ligand 1), CXCL13 (C-X3-C Motif Chemokine Ligand 13) [51], and CCL-2 (C–C Motif Chemokine Ligand 2) productions were positively associated with brain metastases [52]. Besides interleukins and interferons, chemokine identification in the cerebrospinal fluid of melanoma patients can also indicate brain metastasis [53]. Nevertheless, further studies and trials are needed to determine the relative accuracy of detection and to characterize the molecular foundation of these cytokines.

### Immune cells in the circulation STIE

Regarding the responses of immune cells to brain metastasis, patients with circulating lymphocytes of baseline counts > 1000/µL had reduced risks of intracranial recurrence compared with those of ≤ 1000/µL. Meanwhile, higher circulating lymphocyte count was also associated with improved intracranial disease control [54]. In addition, a retrospective observational study of 210 patients with NSCLC found that the neutrophil-to-lymphocyte ratio was positively correlated with NSCLC brain metastasis (OR: 1.12, 95% CI: 1.01–1.23, p = 0.025) [55]. One study investigated the fused immune and tumor cells, and found that an increased number of the fused cells in peripheral blood is associated with worse survival of patients with pancreatic ductal adenocarcinoma [56]. Patients with lung cancer brain metastasis (n = 34) had increased PD-L1^+^ peripheral monocyte, the MDSC abundance (CD33^+^, CD11b^+^, HLA-DR^low^) and Treg percentage (CD3^+^, CD4^+^, CD25^+^, FoxP3^+^) compared to early-stage pre-metastatic patients (n = 15). Patients with elevated PD-L1^+^ peripheral monocytes had less reactive T cells and worse survival [57].

Furthermore, PD-1/CTLA-4 blockade also dramatically increased the trafficking of CD8^+^ T cells to the brain in the melanoma brain metastasis mouse model [58]. CD14 + HLA-DR negative or low monocytic MDSCs were significantly increased in the peripheral blood of patients with brain recurrence compared to those with radiation necrosis after stereotactic radiosurgery in brain metastasis patients. In contrast, expression of Vanin-2 on circulating CD14 + monocytes were decreased in patients with brain metastasis compared to that of patients with radiation necrosis [59]. (Table 1) Radiation and systemic immunotherapy combination treatments produced stronger systemic anti-tumor immune responses by increasing the numbers of activated, cytotoxic CD8^+^ T cells in melanoma mouse models [60].

**Table 1:** Studies on circulating immune cells in patients with brain metastasis

Primary Cancer	Pre-Treatment	Number of patients	Biomarker	Method	Sample type	Cut-off	HR/OR	95%CI	P value	Follow- up	Prognosis	References
Melanoma	SRS, ICI	99	Lymphocyte count	Lymphocyte count	Blood	1000/µL	0.46	0.23–0.94	0.03	15.5 mos	NR	[54]
NSCLC	No	210	NLR	Blood count	Blood	NR	1.12	1.01–1.23	< 0.05	NR	NR	[55]
NSCLC	Surgery	34	MDSC	FACS	Blood	Median value	NR	NR	NR	NR	worse OS	[57]
NSCLC	Surgery	34	Treg	FACS	Blood	Median value	NR	NR	NR	NR	worse OS	[57]
Multiple	SRS	22	MDSC	FACS	Blood	NR	NR	NR	NR	NR	RN	[59]

*No* no treatment, *NSCLC* Non-Small Cell Lung Cancer, *NR* not reported, *HR* hazard ratio, *OR* odds ratio, *FACS* Fluorescence-Activated Cell Sorting, *NLR* neutrophil-to-Lymphocyte, *MDSC* Myeloid-derived suppressor cells, *SRS* stereotactic radiosurgery, *ICI* immune checkpoint inhibitor, *RN* radiation necrosis, *mos* months

### Immune biomarkers associated with TIME of the brain metastatic lesion

**Fig. 2:**
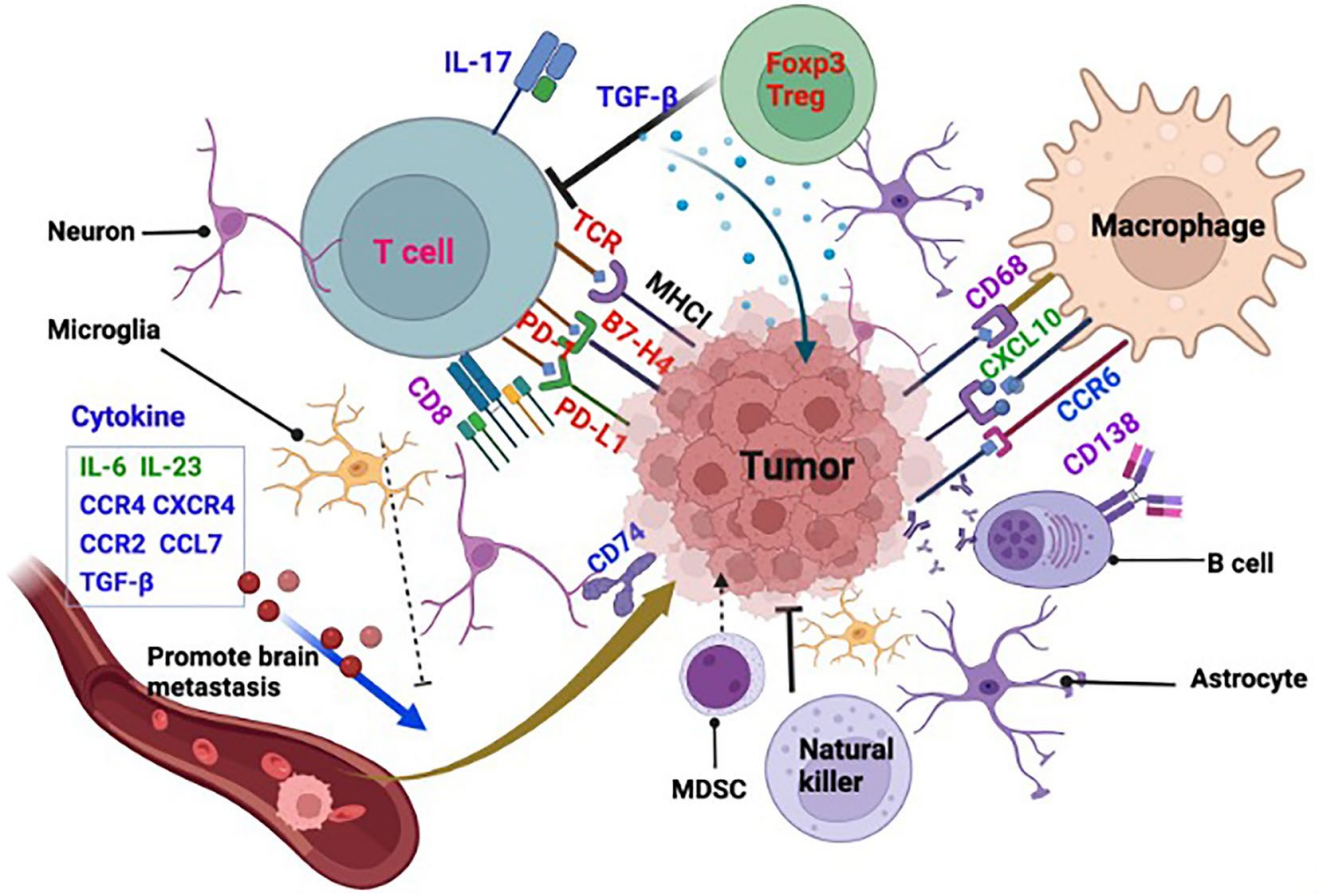
Illustration of Brain TIME Immune Biomarkers. Published biomarkers are shown. Red color biomarkers are reported to be negative association with survival in patients with brain metastasis. Blue color biomarkers are the risk factors for brain metastasis. Purple colored are immune suppressive factors associated with favorable outcomes of brain metastasis in patients. Green color biomarkers are proved to enhance the risk of brain metastasis in mouse model. *TIME* tumor-immune microenvironment, *TCR* T cell receptor, *Foxp3* forkhead box P3, *Treg* Regulatory T cells, *MDSC* Myeloid-derived suppressor cells

Immune perturbations of the metastatic site also play important roles on the prognosis and or their responses to the treatment in the TIME. Revolutionized single cell analysis technology has revealed an abundant and complex immune cell landscape in the brain metastatic region, which includes lymphocytes, macrophages, dendritic cells, innate lymphoid cells, monocytes, MDSC and even granulocytes [61] (Fig. 2).

### Immune cells in the brain TIME

#### Tumor infiltrating lymphocytes (TILs)

As the two major types of lymphocytes in the adaptive immune system, B cells and T cells provide protection against cancer while maintaining immune self-tolerance. High amounts of TILs were negatively correlated with renal cancer smaller brain metastases size [62, 63]. Low TILs percentage, divided by the median percentage of tumor infiltrating lymphocyte, displayed a shorter overall survival than those with high TILs (p = 0.131) following the initial brain metastasis diagnosis. Besides, low total TIL counts were associated with significantly shorter overall survival rates only in breast metastasis brain samples (p = 0.04) [64]. These results reveal that patients with increased TILs tend to display favorable outcomes. The detailed literature on the subtypes of these TILs is shown in Table 2, various subtypes of T cells such as CD8^+^ T cells, CD3^+^ T cell are significantly correlated with overall survival.

**Table 2:** Lymphocytes as biomarker for prognosis and or prediction in brain metastasis

Primary Cancer	Pre-Treatment	Number of patients	Biomarker	Method	Sample type	Cut-off	HR	95% CI	P value	Median follow-up (months)	Prognosis	References
SCLC	/	32	CD45RO + TIL	IHC	Tissue	sparse /scattered	NR	0.000–26.14	0.007	11	**longer survival**	[66]
NSCLC	ICI	25	stromal CD8 + T	IHC	Tissue	Mean value of CD8 TIL infiltration	NR	NR	0.031	NR	**longer survival**	[74]
Lung cancer	Surgery	15	CD8 + T cells	IHC	Tissue	0.10%	NR	NR	NR	NR	**longer survival**	[34]
Breast cancer	Surgery,STI, WBI	89	TIL	IHC	Tissue	0.10%	NR	NR	0.13	13	**longer survival**	[64]
Multiple cancers*	NIL	26	CD3 T-cell density	IHC	Tissue	Immunoreactive cells	NR	NR	0.016	5.8	**longer survival**	[68]
Multiple cancers*	Surgery,STI, WBI	223	CD8 intratumoral expression	IHC	Tissue	0.10%	NR	NR	0.07	32	NR	[69]
Lung cancer	Surgery + TKI	12	TCR clonality	NGS	Tissue and blood	TCR clonality diversity	0.175	NR	p < 0.001	NR	worse OS	[31]
Lung carcinoma	Surgery	33	higher TCR clonality	NGS	Tissue and blood	higher TCR clonality	NR	NR	NR	NR	NR	[31]
Lung carcinoma	Surgery	33	TPS	NGS	Tissue	Positive	0.16	NR	p = 0.001	NR	NR	[31]

*NR* Non report, *FACS* Fluorescence-Activated Cell Sorting, *SRS* Stereotactic radiosurgery, *OS* overall survival, *ICI* Immune checkpoint inhibitor, *SCLC* small cell lung cancer, *TIL* tumor infiltrating lymphocytes, *IHC* Immunohistochemistry, *TCR* T-cell receptor, *STI* stereotactic irradiation, *WBI* whole-brain irradiation, *TKI* Tyrosine kinase inhibitors

#### T cells

AS critical effector lymphocytes, T cells can adopt a wide spectrum of phenotypes, ranging from highly cytotoxic effector T cells (mainly CD8^+^ T cells) to immune-modulating T cells (CD4^+^ T cells) as well as the T regulatory cells in brain metastasis from various cancer types [21, 46, 65–67]. Increased peritumoral T cell density indicated by the CD3 expression was significantly associated with prolonged survival time (median 8.1 months *versus* 5.2 months, p = 0.016) [68].

*CD8*^+^ *T* *cells****:*** Significant CD8 peritumoral expression was found in 68.6% of brain metastasis and in 87.7% of primary tumors. The expression level of CD8 was concordant between primary and metastatic tumors in 73.3% of cases [69]. Notably, the numbers and density of CD8^+^ T cells were significantly lower in brain metastases than in primary lung cancers [70, 71]. It was found that the decreased percentage of cytotoxic CD8^+^ T cells may be caused by the increased numbers and patterns of IDO-expressing tumor cells in melanoma brain metastases [72]. Furthermore, the CD3/CD8 ratio showed an independent and strong impact in brain metastasis patients. The evidence for the anti-tumor role of the CD8^+^ T cell subset are compelling, as reflected by a series of prognostic and treatment responses analyses [73]. Intratumoral CD8^+^ T cells percentage was marginally associated with better prognosis (p = 0.07) for lung cancer brain metastasis [74].

#### CD4^+^ T cells

The CD4^+^ T cells can exert anti-tumor or pro-tumor effects in an environmental dependent manner according to differentiations into T helper cells or T regulatory cells [75]. Distinct CD4^+^ T cell subsets are associated with either good or poor clinical prognoses [16]. Enhanced inhibition of Th1 was also observed in lung adenocarcinoma brain metastases compared with primary tumors [76]. Patients with brain metastatic lung carcinoma demonstrated increased Treg percentage compared to early-stage pre-metastatic patients and healthy controls and was associated with worse survival [57]. In contrast, it has been reported that the number of T helper 17 cells increased in patients with brain metastases from lung cancer (including both SCLC and NSCLC) [77]. These contradictory results may partially explain why cancer immunotherapy leads to favorable outcomes in only some but not all brain metastasis patients [78, 79].

#### T Cell Receptors (TCRs)

Apart from the different phenotypes of T cells that were found in brain metastases, T cell expansion as indicated by the T-cell receptor repertoire analysis also displayed differential patterns between the brain metastasis lesions and primary tumor sites. TCRs expressed on all kinds of T cells' surface mediate antigen recognition, T cell responses, T cell expansion and present another element of diversity within T cell populations. Longitudinal immune cells profiling facilitates the characterization of the immune TME in brain metastatic lesions and monitors the evolution of the cancer immune response over treatment courses [80]. Mansfield et al. found that there was a significant discrepancy in the number of unique T cell clones in brain metastases compared to paired primary cancers, with brain metastases tissue samples having higher non-synonymous mutation burdens than primary lesions [81]. Higher TCR clonality was associated with prolonged OS in EGFR-treated patients but worse outcomes in non-EGFR-treated patients [31]. Moreover, Kudo et al. indicated that brain metastases exhibited lower T cell and elevated macrophage infiltration compared with primary tumors, while TCR repertoires were largely shared between paired brain metastases and primary tumors [76]. In contrast, stronger oligoclonal T cell expansion and higher TCR clonality was demonstrated in brain metastases than in primary lung tumors.

Taken together, although T cell expansion was limited in the brain metastasis sites, the T cell clones were similar in different metastasis regions. It may be possible that the antigens that activate T cell expansion mostly come from the primary tumor sites rather than from the brain metastasis sites, which are separated by the BBB [82–84]. The phenotypic and clonal diversities of T cells found in brain metastases from different cancer types display distinguished levels when compared to their respective primary tumors, and exhibit potentials as biomarkers in brain metastases.

#### B cells

B lymphocytes also play an important role in coordinating humoral immunity of the adaptive immune system [85]. When a naive or memory B cell is activated by an antigen, it proliferates and differentiates into an antibody-secreting effector cell (plasma cell), presents antigens and secretes cytokines to regulate other immune functions [86]. B cells which have been recruited to the brain during an immune reaction, most likely in response to injury, could persist for an extended period and eventually transform while residing in the brain. On the other hand, B cells could be transformed outside the brain before entering the brain [87]. Fewer B cells were observed in the matched brain metastasis lesions as compared to the primary tumor in triple negative breast cancer [88]. The involvement of B cell in the progression of brain metastasis was supported by the evidence that the metastatic burden was increased because of B cell depletion in preclinical models [89]. In addition, a study from 13 patients found that patients' tumor tissues that expressed high levels of CD138 plasma cells had a statistically significant improvement in overall survival compared to low levels of CD138 [90]. However, further studies are required to establish the exact roles of the different subsets of B cells in brain metastasis.

#### Natural Killer (NK) cells

NK cells are large granular lymphocytes that play an important role in antitumor immunity [91]. Upon activation, NK cells induce target cell apoptosis through contact-dependent cytotoxicity. Metastatic tumor cells of distinct developmental tumor stages exhibited differential sensitivities to NK cell surveillance in patient and mouse models in a SOX9-dependent resistance mechanism-dependent manner and displayed context-dependent adaptation and immune evasion as demonstrated by scRNA-seq [92]. Although clinical trials on combination therapies involving NK cells might enhance the effectiveness and/or overcome brain tumor immune escape mechanisms (NCT02271711, NCT01804634 and NCT02100891), both clinical and pre-clinical evidence in brain metastasis required further investigations.

#### Tumor associated macrophages (TAMs)

The brain tumor-associated macrophage (TAM) population consists of cells originating from resident microglia (TAM-MG) and cells of monocytic origin, i.e., monocytes and monocyte-derived macrophages (MDM) [93]. They are the predominant immune population that critically influence the progression and outcome in the brain by metastatic melanoma, breast, and colon cancer [94]. At the beginning of the metastasis process, the TAM-MG can eliminate the tumor cells as the host defense mechanism by the phagocytosis process, interaction with other brain resident cells and inducing the expression of the major histocompatibility complex I (MHC I) [95, 96]. During the cerebral or cerebellar tumor formation, cancer cells hijack several mechanisms to polarize microglia to the activated status with and recruit the peripheral MDM, which expressed MHCII, PD-L1, IL-1A and TNF in the brain metastasis [97, 98]. Both the polarized microglia and MDM-TAM represent the most abundant stromal cell types in instigating and supporting brain metastases. TAMs demonstrate phenotypic plasticity and exist in two broadly defined polarization states, namely M1 and M2 [99]. As classically activated, M1 macrophages promote an inflammatory response against tumor cells by releasing the IL-12 and IL-19 that contribute to the classic T helper cell-1 responses, while M2 macrophages contribute to immunosuppression [100, 101]. They can also ameliorate the anti-tumor immune responses by reducing the expression of key molecules involved in T-cell co-stimulation (ie. CD80 or CD40), impairing antigen presentation [102]. The polarity of microglia promoted the brain metastasis by increasing the secretion of IGF-1 and CCL20, C-X-C motif chemokine 5 (CXCL5), CXCL8 and interleukin (IL)-6 were increased in the setting of brain metastasis. These chemokines recruit immunosuppressed neutrophils into the metastatic niche [103, 104].

The diversity of the TAM pool is regulated by the origin of the primary tumor in human brain metastasis. While the density of CD68^+^ TAMs was similar in primary renal cancer and brain metastases, the CCR2-positive TAMs (proinflammatory type) were more frequently expressed in brain metastases than in primary renal cancer [105]. Consistently, vast expansion of M2 type TAMs was found in the brain metastasis lesions of NSCLC patients [106], which contributed to the immunosuppressive microenvironment [103]. scRNA-seq further demonstrated that the TAMs in primary lung tumors and distant metastases mainly propagated from MDM-TAMs that were ontologically different from tissue-resident macrophages [107]. The MG-TAMs were more immunosuppressive than their MDM-TAMs [108]. M2 macrophages were paramount in NSCLC brain metastases, as they promoted tumor progression and immune evasion [71]. A heterogeneous distribution of macrophage states representing a continuum of inflammatory and immunosuppressive/pro-tumor phenotypes were also found in brain metastatic lesions [95]. A scRNA-seq in brain metastasis mouse model under treatment-naive conditions or WBRT found that MG-TAM represents a more homogeneous population compared with MDM-TAM. Higher expression of pro-inflammatory mediators in MG-TAM including Cxcl13, Ccl3, Ccl4, and C1qb and the microglial marker Tmem119 and Hexb was increased after radiotherapy. These inflammatory genes were positively associated with the antigen presentation, which promotes immune responses [109, 110].

These findings illustrated the dynamics and importance of the TAM pool in the brain metastasis. The relative contribution of each TAM population to the brain metastasis is influenced by the metastasis tumor cells.

#### MDSC

MDSCs are heterogeneous population of immature myeloid cells that can be typically distinguished as polymorphonuclear and monocytic MDSC. MDSCs can promote angiogenesis, tumor cell invasion and metastases through releasing the variety of soluble factors and inhibiting the T cell activity [111]. The infiltration of MDSCs (CD11b^+^Gr1^+^) was greater in dural metastases than in brain parenchymal lesions [112]. In both mouse and human brain metastasis cases, the infiltration of MDSCs (CD11^+^Gr-1^+^ for mice and CD14^−^CD15^+^HLA-DR^low^ for human) was observed at high levels in the tumor tissues. Mice with genetic deficiency for CCR2, a receptor for CCL2, exhibited low penetration of brain metastases along with reduced infiltration of MDSCs [113]. The presence of Gr-1^+^CD11b^+^ MDSCs in the premetastatic brain can contribute to the formation in the breast cancer brain metastasis [114]. In sum, the MDSCs can promote cancer to brain metastasis.

Collectively, the immune cells reflect the diverse immune responses, which suggested that the functional status of TILs and the tumor inflammatory milieu are all key biological factors for biomarker identification in brain metastases lesions. These studies support a conceptual framework in which specific immune cells in the TME may be best suited for engineering tumor-specific immune cells. Such tactics have the potential to improve the recruitment of effector T cells to tumors during adoptive cell therapy and to promote the infiltration of chimeric antigen receptor (CAR) T cells. For example, clinical trials (NCT03696030) are enrolling patients to study the efficiency of HER2-CAR T cells in patients with recurrent brain or leptomeningeal metastases.

### Immune modulating biomarkers associated brain TIME

#### Immune checkpoints

##### PD-1

The immune checkpoint receptors in immune cells such as programmed cell death receptor 1 (PD-1), have been implicated in immune dysfunction and brain tumor-mediated immune suppression [115]. A comparative analysis in nine patients with extracranial melanomas and matched intracranial metastases found that the expression of PD-1 was conserved in between extracranial and intracranial tumoral [116]. Survival after craniotomy was also positively correlated with PD-1 expression on the surface of TILs in breast cancer brain metastases (hazard ratio (HR) = 0.3, p = 0.003) [117].

##### CD74

CD74 is a HLA class II-chaperone molecule involved in antigen presentation [118]. A study from 236 human brain metastases patients found that the CD74 expression on tumor cells was a strong positive prognostic marker in brain metastasis patients [119]. Another study from 49 patients found that the CD74-ROS1 rearrangement group have a higher rate of brain metastases (p = 0.020) [120]. CD74 knockdown in vitro leads to a reduction of HLA class II peptidome complexity, which promotes the immune evasion of the brain metastasis cells [119].

##### VISTA (V-domain Ig suppressor of T cell activation)

VISTA is a ligand with homology to the extracellular domain of B7 ligand PD-L1. The expression of VISTA was found to be upregulated on the myeloid antigen presenting cells, which inhibited T-cell proliferation and cytokine production [121, 122]. VISTA blockade along with anti-PD-L1 reduced brain metastasis outgrowth and additionally, led to reduced TILs density. Besides, VISTA^+^PD-L1^+^ brain-myeloid cells displayed immunosuppressive properties that promote brain metastasis outgrowth [123, 124]. The clinical perspective in understanding the association of VISTA expression in the TIME compartment of brain metastasis may reveal new promising immunotherapies targets.

#### Immune checkpoints ligands

##### PD-L1

Dysregulation of immune checkpoint ligands, such as programmed death-ligand 1 (PD-L1), have been implicated in immune dysfunction and brain tumor-mediated immune suppression [115]. Negative immune checkpoint regulator, the inhibitory costimulatory molecule of programmed death ligand 1 (PD-L1) plays a critical role in adaptive cellular immunity [125]. The upregulated expression of PD-L1 on the surface of various types of cancer cells inhibited T-cell activation and differentiation.

NSCLC brain metastases patients with elevated peripheral monocyte PD-L1 had less reactive T cells and worse survival [57]. Postoperative SCLC specimens were immunoassayed with the SP142 antibody against PD-L1. The median survival time was longer in the PD-L1 positive group (46.4 *versus* 28.5 months, p = 0.002). The 3‐year risk of brain metastasis in the PD‐L1 positive group was lower than that in the PD‐L1 negative group (24.1 *versus* 48.4%, p = 0.046). PD-L1 was an independent factor for overall survival (HR = 0.485, p = 0.011) and brain metastasis (HR = 0.335, p = 0.024) [126]. The PD-L1-positive brain metastasis group had a significantly shorter brain-specific disease-free survival than the PD-L1-negative resected brain-metastatic NSCLC brain metastasis group (p < 0.05) [127]. Patients with tumors showing PD-L1 expression of at least 1% in stromal or immune cells had a longer overall survival than those with PD-L1 less than 1% (median overall survival 11.0 months [95% CI 7.8–NR] *versus* 2.7 months [1–NR], p = 0.031) [128] (table. 3). It was found that sex, age, and the status of brain metastases were predictive parameters for the treatment responses for cancer patients after anti-PD-1/PD-L1-based therapy [129].Patients with lower expressions of PD-L1 or p53 proteins, who only underwent surgical treatment for brain metastases may have worse prognosis [34].

**Table 3:** Immune-checkpoints and ligands in prognosis prediction in brain metastasis

Primary Cancer	No	Treatment	Biomarker	Method	Sample type	Cut-off	HR	CI	P	Follow up	Prognosis	References
Breast Cancer	84	No	PD-1	IHC	Tissue	0.10%	0.3	NR	< 0.003	21 mos	Longer OS	[117]
Multiple cancer*	236	No	CD74	Microarray	Tissue	Score > 20	NR	NR	0.001	4166 days	Longer OS	[119]
Breast Cancer	20	NO	Vista	IHC	Tissue	NR	NR	NR	NR	NR	NR	[123]
SCLC	32	No	PD-L1	IHC	Tissue	5%	NR	NR	0.002	NR	Longer OS	[126]
NSCLC	34	ICI	PD-L1	FACS	Tissue/blood	Median	NR	7.8–NR	0.003	1200 days	Worse OS	[127]
NSCLC	49	No	B7-H4	IHC	Tissue	Median	NR	NR	0.002	14 mons	Worse OS	[131]

*No*: Not previous treatment, *NR* Non report, *ICI* immune check-point inhibitor, *SCLC* small cell lung cancer, *IHC* Immunohistochemistry, *NSCLC* non-small cell lung cancer, *FACS* Fluorescence-Activated Cell Sorting, *OS* Overall Survival. Multiple cancer*: brain metastasis of melanoma (n = 96), NSCLC (n = 56), breast carcinoma (n = 31), renal cancer (n = 18), small cell lung cancer (n = 8), colon carcinoma (n = 10), not specified carcinoma (n = 8) and rare tumors (n = 9). *P* P value, *mos* months

##### B7-H4

B7-H4 is a ligand in the B7 costimulatory family that negatively regulates T cell immune response and promotes immune escape by inhibiting the proliferation, cytokine secretion, and cell cycle of T cells [130]. Median overall survivals were also significantly shorter in patients with higher expression of B7-H4, an immune costimulatory protein, in NSCLC brain metastases [131] (Table 3).

These results suggest that immune checkpoint ligands such as PD-L1 can act as prognostic biomarkers in brain metastases (Table 3). All these biomarkers have variable predictive accuracy for anti-PD1 or anti-PDL1 therapy efficacy across various histology. Besides, the immune checkpoint inhibitors (ICIs) can be the drug targets and improve the prognosis in the brain metastasis patients by boosting the immune response. Indeed, in a recent retrospective analysis of five patients with new or progressing brain metastases from NSCLC treated with PD-1 blockade, an objective response was observed in two patients and persisted for greater than 6 months, suggesting a possible role for anti-PD-1 therapy in treating brain metastases [132]. A meta-analysis including a total of 1330 ICI-treated brain patients indicated that the 6-month survival rate and progression free survival were 0.67 (95% CI: 0.59–0.74) and 0.36 (95% CI: 0.24–0.49), respectively [133]. Collectively, these preclinical and clinical evidence implicates that targeting of these immune checkpoints presents a new opportunity for clinical management of brain metastasis.

### Immunomodulating cytokines in brain TIME

Cytokines are a diverse family of low-molecular weight proteins involved in the communication between cells. They exhibit complex roles in immunity, host defense, inflammation, as well as in tumor immunobiology through various autocrine, paracrine, and/or endocrine mechanisms [134]. The major sub-groups of cytokines include interleukins [135], interferons, colony-stimulating factors, chemokines as well as tumor necrosis factors, and they are produced either as secreted or membrane-bound protein during the process of brain metastasis [136]. This article is not intended to provide detailed information about cytokines in brain metastasis, as this is covered in other recent excellent reviews [135], but rather highlights cytokines that are associated with predicting the brain metastasis and prognostic outcomes in patients with brain metastasis.

Increased IL-6 concentration enhances the possibility of breast cancer brain metastasis by increasing the permeability of BBB [52]. Elevated levels of CCR4 is another predictive marker for melanoma brain metastasis in an analysis of melanoma brain metastasis cell lines [137]. CCR4 is significantly higher in paired clinical specimens of melanoma metastases than in samples of primary tumors from the same patients. Functionally, the human melanoma cells over-expressing CCR4 were more tumorigenic and caused a higher load of brain metastasis in mouse model [138]. CD37, IL-23A, tumor necrosis factor-a (TNF-a), CD34, CD48, and CD27 were downregulated in lung cancer brain metastasis through TCGA analysis compared with the lymphoid node metastasis [139]. Furthermore, brain metastasis cases exhibited significantly lower expression of interleukin 13 receptor alpha2 (IL-13Ralpha2) than non-metastasis cases in five cases [140]. Astrocytes can be reprogrammed by human brain-metastasizing melanoma cells to express pro-inflammatory factors, including the cytokine IL-23, which was highly expressed by metastasis-associated astrocytes in vivo. IL-23 was sufficient to increase melanoma cell invasion, and astrocyte-derived IL-23 in facilitating the progression of melanoma brain metastasis [141]. A study of 246 patients with metastatic renal cancer found that CCR2 and CCL7 expressions were upregulated in brain metastases compared with primary tumors [105]. In contrast, the CCL2, CCL19, C-X-C motif chemokine receptor 6 and C–C motif chemokine receptor 2 were down-regulated in lung cancer brain metastasis [139]. CXCL10 was upregulated in metastasis-associated astrocytes in mice and humans and was functionally important for the chemoattraction of melanoma cells [142]. Overexpression of CXCR4 protein was observed in 29 (90.6%) non-small cell lung cancers and in all (100%) brain metastatic tumors and was significantly higher in the primary brain tumors than that in the primary tumor sites (p < 0.000). The 3- and 5-year cumulative survival rates of patients with solitary brain metastasis of lung cancer were 21.9 and 12.5%, which are significantly lower than the corresponding survival rates of group patients without distant metastasis (p = 0.005) [143] (Table 4).

**Table 4:** Cytokine expression in brain metastasis

Primary Cancer	No	Model	Biomarker	Method	Sample type	Endpoint	Reference s
Breast cancer	NA	Mouse	IL-6	ELISA	Tissue	Enhanced possibility of brain metastasis	[52]
Melanoma	12	Patient	CCR4	IHC	Tissue	Enhanced possibility of brain metastasis	[138]
Melanoma	NA	Mouse	IL-23	Human Cytokine Array Panel	Cell lysate	Enhanced possibility of brain metastasis	[141]
NSCLC	32	Patient	CXCR4	IHC	Tissue	Worse survival	[143]

*NA* Not applicable, *NR* Non report, *IHC* Immunohistochemistry

Cytokines and chemokines are other promising immune biomarkers as well as drug targets in brain metastasis (Fig. 1). Despite some encouraging preclinical studies, cytokines or chemokine-targeted therapies for the treatment of patients with brain metastases are still far from reach. Using chemokine targeting agents combined with existing cancer therapies might show synergistic therapeutic effects. Additional experiments shall be conducted to explore the underlying mechanism how the levels of these cytokines are modulated.

## Immune related biomarkers in the CSF STIE

### Immune cells in the CSF

CSF can be a source of biomarker testing with minimally invasive procedures. Rubio-Perez et al. used single-cell RNA sequencing combined with T cell receptor genotyping in cerebrospinal fluid and found that amongst the immune infiltrates, specifically CD8^+^ T cell infiltrates, identical T cell receptor clonotypes are detected across brain lesions and CSF [80]. These results suggest the pattern of T cell clones in CSF was similar to that of the brain lesions. Studies regarding the functional interactions between the CTCs with immune cells in the circulation are also emerging. A single cell analysis from 50 surgical specimens and corresponding CSF displayed the phenotypic diversity of lymphocytes in brain metastases, which differed from the primary tumor and with different cancer types. When analyzing the CSF upon tumor resection, cytotoxic lymphocyte and naive T cells increased and tumor associated macrophages decreased in abundances, while CD8^+^ T and NK cell levels were similar in the tumor and in CSF [83]. The monocytes tend to transit from monocytes to macrophages M2-subtype in CSF samples from lung cancer brain metastasis [80]. Clearly, although CSF testing of immune cells has a potential to better understand the brain metastasis in cancer, large sample size and more studies are required.

### Immune modulating factors such as cytokines in the CSF

Many immune modulating factors can be detected in CSF, but study on this topic is limited. A study of individual CSF samples from 22 patients with melanoma brain metastases and 5 disease-free controls found that Chemokine CCL22 and cytokines IL-1α, IL-4, and IL-5 were reduced in most samples, whereas a subset of molecules including CXCL10, CCL4, CCL17, and IL8 increased expression [144]. Further, analysis of clusters identified within the melanoma patient set comparing patient outcome suggests that suppression of IL-1α, IL-4, IL-5, and CCL22, with concomitant elevation of CXCL10, CCL4, and CCL17, may correlate with more aggressive development of brain metastasis [144]. There are no studies on transforming growth factor-β1 (TGF-β1), metabolites like IDO metabolic products as well as PD-1 and ligands measurements on the CSF. Clearly more studies are needed on this topic.

## Molecular immune modulators associated with TIME and STIE

As shown in Figs. 1 and 2, STIE and TIME establish a connection through immune cells and metastasis tumor cells and secreting immune modulating factors. In addition to the above discussed cytokines, the following sections will discuss about TGF-β1 and IDO metabolites [61].

### TGF-β1

TGF-β1 is described as an epithelial-mesenchymal transition master inducer in the metastatic process both in the TIME and STIE. In the TIME of primary tumor sites, the TGF- β1 rs1800469 polymorphism was found to be a predictive biomarker for the risk of developing brain metastasis in patients with NSCLC [145]. In addition, the single nucleotide polymorphisms (SNPs) of SMAD5 in the TGF-β1 signaling pathway, such as the GG genotype of SMAD6: rs12913975 and TT genotype of INHBC: rs4760259, are associated with a higher risk of brain metastasis in patients with NSCLC [146]. Moreover, a multicenter study of fourteen functional SNPs in the TGF-β1 pathway in 166 patients with NSCLC found that BMP2:rs235756, SMAD9:rs7333607, SMAD3:rs12102171 and SMAD4: rs12456284 were significant predictors of survivals [147].

In the STIE, TGF-β1-mediated exosomes can enhance the BBB permeability to promote the brain metastasis process [148]. Our group has previously demonstrated that TGF-β1 in plasma may be a biomarker for tumor progression and survival in lung cancer [149]. Although anti-metastasis therapies targeting the modulation of TGF-β level and regulatory signaling molecules have been developed, the clinical outcomes do not meet expectations [150]. Further work aiming to reduce the level TGF-β both in the TIME and STIE may be beneficial.

### Immunometabolites

The presence of cancer cells alters the metabolic activities of the normal tissues, metabolic heterogeneity and plasticity in TIME [151]. It is believed that “immunometabolites” like succinate, itaconate, acetyl-CoA, and 2-hydroxyglutarate serve as signal transducers that regulate immune cell function and metastasis [152]. Immune cells rely on specific metabolic pathways for activation and differentiation to responses to tumor cells [153]. For example, it has been shown that blocking glutamine metabolism enhances antitumor immune T responses[154], which can inhibit brain metastasis [155]. Lactate secretion by tumor cells can promote breast cancer brain metastases by inhibiting the cytotoxic activity from natural killer cells [156]. The colonization of tumor cells at distal sites requires metabolic adaptation based on the distinct nutrient availability in the new TIME compared with the primary tumor site. It was found that LEF1 facilitates metastasis by improving the antioxidative capacity of epithelial breast cancer cells, during colonization of the brain parenchyma [157]. Preclinical studies from mouse model showed that targeting of xc–anionic amino acid transporter (xCT) can also promote breast cancer brain metastases by impairing the NK cell activity. Accordingly, the xCT expression is significantly higher in brain metastatic samples compared to primary tumors in breast cancer patients [158].

Notably, the enzyme Indoleamine-pyrrole 2,3-dioxygenase (IDO), an enzyme that metabolizes tryptophan to kynurenine, was found to be upregulated in some colorectal carcinoma brain metastases patients [159]. In the TIME, the release of kynurenine in this process enhances the Treg differentiation and promotes the differentiation of antigen presenting cells to the immunosuppressive phenotypes [160]. Other metabolic by-products resulting from IDO activation, including 3-hydroxyanthranilic acid, also inhibits T cell and natural killer cell proliferation and function [161]. In the STIE, the functions of kynurenine was also closely associated with the immune-suppressive status of patients[162]. Additionally, the plasma or serum concentrations of kynurenine was also positively associated with the disease progression and metastasis status in hepatocellular carcinoma [163], lung cancer [164, 165]. Further work is required to investigate the roles of IDO associated metabolites in brain metastasis.

Collectively, the monitoring of metabolite changes and primary indicator of systemic responses is a promising approach to diagnose the brain metastasis process. Tissue metabolomic investigations enable deeper insights into aberrant immune metabolism occurring at the site of disease pathogenesis [166] and biological liquid metabolomics from plasma, serum, saliva, CSF has supported the widespread use of metabolites as biomarkers in brain metastasis. With advantages of high throughput, high sensitivity, and high accuracy [167], metabolomics for biomarker discovery offers potential advantages in sensitivity and specificity in biomarker discovery in this field.

## Summary of current status, limitations and future direction

In summary, the immune biomarkers may play important roles in risk prediction, prognosis, and treatment reponses in brain metastasis. Mutated immune related genes in the primary tumor TIME may increase the risk of brain metastasis in patients and act as the potential drug targets for early intervention. The immune cells in the brain TIME are significantly associated with prognosis of patients with the brain metastasis. Enrichment of CD8^+^ T cells is associated with favorable clinical outcomes. The Treg cells are often associated with poor prognosis of the patients, while the T-helper cells are associated with enhanced immune responses. The myeloid cells in brain metastases are diverse in terms of composition and transcriptional profiles. As tissue resident macrophages, the microglia could be the first line of defense from brain tumor cells and be hijacked to induce immune suppressive environments. The macrophages from blood tend to switch to type 2 macrophages during brain metastasis process. The MDSCs can express immune inhibitory factors that promote brain metastasis. Additionally, the immune checkpoints act as prognostics and predictive biomarkers both at the baseline level and after treatment, such as immunotherapy. The levels of TIME cytokines and chemokines can predict brain metastasis because they can increase the permeability of BBB and recruit the immune suppressive cells and tumor cells. Furthermore, the immunometabolites can regulate the behaviors of immune cells and then modulate the brain metastasis. Additionally, the dynamic changes of immune biomarkers at multiple levels can be identified as biomarkers for brain metastasis.

However, the current knowledge on immune biomarker for brain metastasis is limited. The studies on this topic have been challenged by tissue sample scarcity, tumor heterogeneity, treatment cofounding factors and the limited number of well-designed clinical studies. Better and more in depth understating of the metastatic process and the immune system opens the door for discovery of successful future biomarkers for brain metastasis [159, 168]. Hence, strategies to overcome the limitations are needed.

For the limitation of tumor tissue, TIME studies of paired brain metastasis and primary tumor [12], and comprehensive testing of circulating STIE or CSF STIE could be good surrogate [12, 169]. STIE components can be tested with both peripheral blood and CSF samples include lymphocyte counts, cytokines and immune checkpoints expression, and immunometabolites as the immune biomarkers in brain metastasis. Properly banked blood and CSF samples can provide full spectrum of STIE immune biomarkers, which may overcome the limitation of tissue scarcity. In-depth knowledge of the STIE immunological signatures across brain metastasis may be a major step forward for immune biomarkers discovery in brain metastasis.

For the issue related to the often low levels of expression and heterogeneity both across different cancer patients and different primary malignant lesions [170], notably, advances in molecular technology such as single cell sequencing are facilitating the detection of extremely low concentrations of an increasing number of different molecules and cell subtypes [171]. This technology allows the dissection of the gene expression at single-cell resolution at low abundance rates of samples and all aspects of cells, which greatly revolutionizes biomarker studies [172]. It has helped and can continue to help characterizing the cellular phenotypes and regulatory mechanisms of various immune and tumor cell types, within both TIME of the brain metastasis and STIE in the circulation [123].

The complexity of treatment modality and lack of comprehensive consideration of single modality or combined effect [173] are also major limitations of the current literature. Clearly, all kinds of treatment including steroid, surgery, radiation therapy, chemotherapy, target therapy, and immune therapy can lead to complex changes of the immune response which can be assessed by immune biomarker testing [174, 175]. The changes of biomarkers in combination therapies are far more complex than in monotherapies as they need to consider not only the actionable targets of individual agents under investigation but also the interactions of the agents in combination. For example, corticosteroids, which is commonly used to relieve the symptoms of patients, can also alter the TIME and STIE, thus the profile of immune biomarkers [176, 177]. The responses to previous therapies, should be investigated to allow for more efficacious therapeutics for patients with brain metastasis [178, 179]. As such, it is difficult to draw conclusions on the absolute effect of single immune biomarkers when patients have undergone different multiple cancer treatments. In addition, examining various clinical factors such as multiple lines of treatments, tumor staging, sizes and number of metastasis when assessing brain metastases, and measurement of the dynamics of biomarkers longitudinally after multi-lines of treatments would be helpful [180], although this also makes it more complicated for biomarkers identification. Compared with single biomarker performance, a machine learning model constructed from the information of different levels of immune biomarkers and the various clinical factors may be more effective to tailor personalized therapies in cancer brain metastasis [181].

Furthermore, well-designed biomarker study requires adequate sample size calculation, a multi-center collaboration model with standardized procedures can help enhance the sample size in biomarker identification [182, 183]. Standardized processing and storage of all tissues, blood and cerebrospinal fluid in proper condition are required for live cell analysis such as lymphocyte subtyping using flow cytometry and single cell RNA sequencing [184, 185].

Overall, the current findings suggest that the dynamic changes of immune biomarkers at multiple levels can be identified as biomarkers for brain metastasis. A comprehensive analysis of large sample sizes and high-quality data from the advanced single cell multi-omics technology by machine learning algorithms may boost the applications of immune biomarkers in brain metastasis. We hope that the novel therapeutic strategies that consider the dynamic changes of the immune biomarkers can facilitate the early intervention of the brain metastasis associated immunological process and thus prevent brain metastasis or avoid worse treatment outcomes.

## Supplementary Information

**Additional file 1: Figure** Illustration of the Biological Process of Cancer Brain Metastasis and Potential Biomarkers. Brain metastasis involves primary tumor cells getting into the circulation (step 1a), surviving in the circulation (step 1b), breaking through the blood brain barrier (step 2) and proliferating in the brain (step 3). The host immune system is involved during each step of this process. This includes (but not limited by) the host immune cells at the primary tumor immune microenvironment (TIME), immune cells (such as lymphocytes, natural killer cells and macrophages) in the circulation, i.e. the systemic tumor immune environment (STIE), the brain TIME, and immune modulating molecules like immune check-point ligands, cytokines and immunometabolites present at the extracellular matrix of the primary tumor TIME, STIE and the brain TIME as well as the brain capillary system. *IDO1* indoleamine 2,3-dioxygenase 1, *Trp* tryptophan

## Data Availability

All data generated are included in this published article or from public sources.

## Abbreviations

BBB: Blood brain barrier
CAR-T: Chimeric antigen receptor T
CHI3L1: Chitinase-3-like-1
CSF: Cerebrospinal Fluid
CTCs: Circulating tumor cells
CTLA-4: Cytotoxic T-lymphocyte-associated protein 4
CXCL: C-X-C motif chemokine
EGFR: Epidermal growth factor receptor
FACS: Fluorescence-activated cell sorting
Foxp3: Forkhead box P3
HLA-G: Human leukocyte antigen G
HR: Hazard ratio
ICI: Immune Checkpoint Inhibitor
IDO: Indoleamine-pyrrole 2,3-dioxygenase
IHC: Immunohistochemistry
IL: Interleukin
IL-13Ralpha2: Interleukin 13 receptor alpha2
Kyn: Kynurenine
MDM-TAM: Monocyte-derived TAM
MDSC: Myeloid-derived Suppressor Cell
MG-TAM: Microglia-derived TAM
MHC I: Major histocompatibility complex I
Mos: Months
NK cells: Natural Killer cells
NLR: Neutrophil to Lymphocyte Ratio
NR: Not reported
NSCLC: Non-small Cell Lung Cancer
NSCLC: Non-small cell lung cancer
OR: Odds ratio
OS: Overall survival
PD-1: Programmed cell death protein 1
PD-L1: Programmed death-ligand 1
PFS: Progression free survival
RCC: Renal cell carcinoma
SCLC: Small cell lung cancer
scRNA-Seq: Single-cell RNA sequencing
SNPs: Polymorphisms
SRS: Stereotactic radiosurgery
Stat3: Signal transducer and activator of transcription 3
STIE: Systemic Tumor Immune Environment
TAM: Tumor Associated Macrophages
TCRs: T cell receptors
TGF-β1: Transforming growth factor-beta1
TIL: Tumor infiltrating lymphocyte
TIME: Tumor immune microenvironment
TMB: Tumor mutation burden
TME: Tumor microenvironment
TNF-a: Tumor necrosis factor-a
Treg: Regulatory T cell
Trp: Tryptophan
VISTA: V-domain immunoglobulin suppressor of T cell activation
xCT: Xc^–^ anionic amino acid transporter
