# Supplementary figures and images for: Immune related biomarkers for cancer metastasis to the brain
Source: Exp Hematol Oncol. 2022 Dec 16;11:105. doi: 10.1186/s40164-022-00349-z (PMC9756766; doi:10.1186/s40164-022-00349-z)

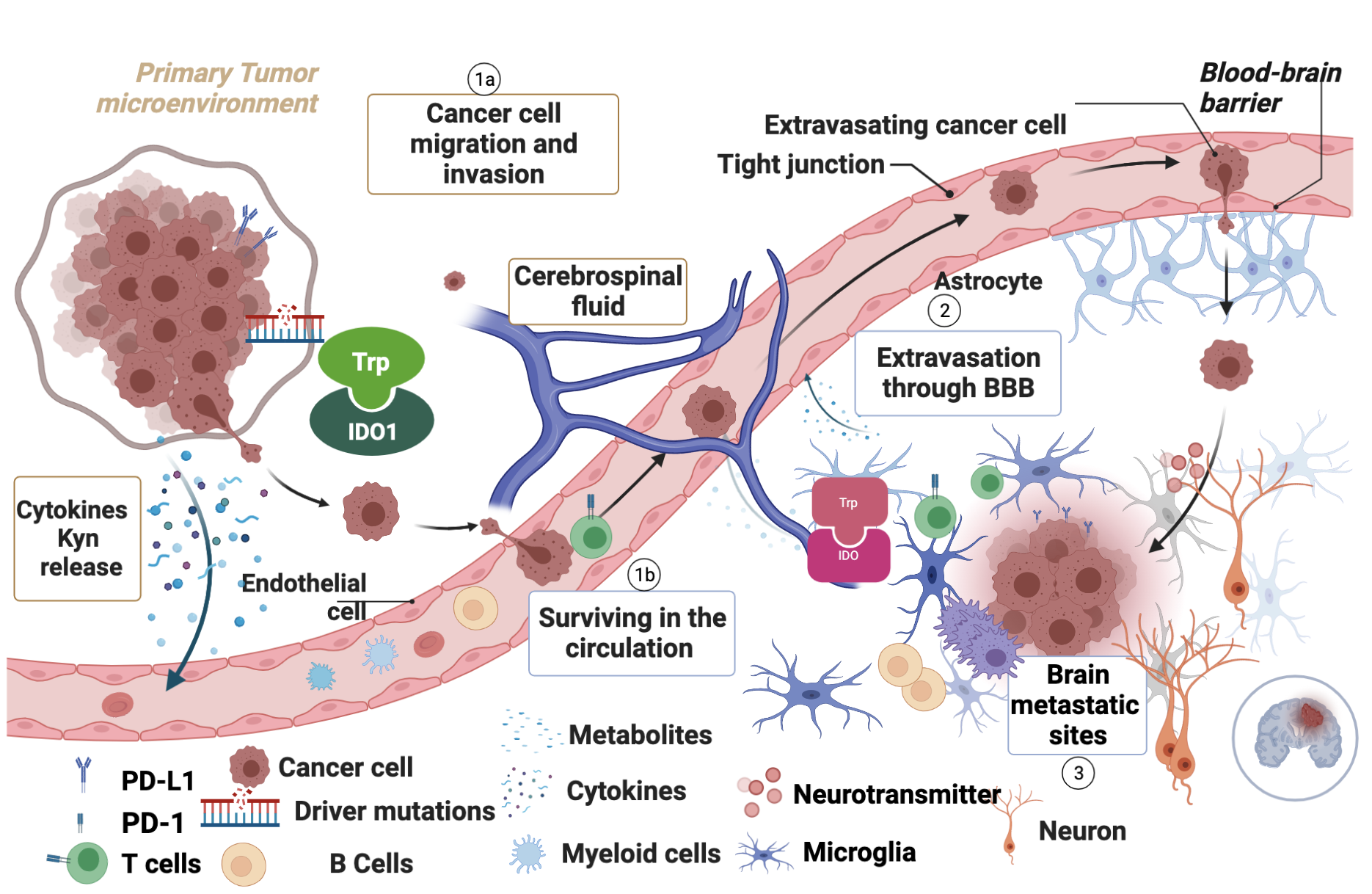

Supplement: Supplementary file 1 — Additional file 1: Figure Illustration of the Biological Process of Cancer Brain Metastasis and Potential Biomarkers. Brain metastasis involves primary tumor cells getting into the circulation (step 1a), surviving in the circulation (step 1b), breaking through the blood brain barrier (step 2) and proliferating in the brain (step 3). The host immune system is involved during each step of this process. This includes (but not limited by) the host immune cells at the primary tumor immune microenvironment (TIME), immune cells (such as lymphocytes, natural killer cells and macrophages) in the circulation, i.e. the systemic tumor immune environment (STIE), the brain TIME, and immune modulating molecules like immune check-point ligands, cytokines and immunometabolites present at the extracellular matrix of the primary tumor TIME, STIE and the brain TIME as well as the brain capillary system. IDO1 indoleamine 2,3-dioxygenase 1, Trp tryptophan [file 40164_2022_349_MOESM1_ESM.png]
